# Syphilis

**Published:** 1917-06

**Authors:** 


### BOOK REVIEWS.

DR. M. F. KREISLE, BOOK REVIEW EDITOR.

SYPHILIS. By Lloyd Thompson, Ph. D., M. D., Physician to the Syphilis Clinic, Government Free Bath House; Visiting Urologist to St. Joseph's Hospital; Consulting Pathologist to the Leo N. Levy Memorial Hospital, Hot Springs, Ark.; First Lieutenant, Medical Reserve Corps, United States Army; member of the American Urological Association, and of the American Association of Immunologists. Pp. 405; 77 engravings and seven colored plates. Philadelphia and New York: Lea and Febiger, 1916.

This work will prove a valuable addition to the library of every practitioner of medicine. It presents in one volume in an orderly and readable manner a comprehensive knowledge of this very prevalent disease accumulated from the literature of all time and from the author's own extensive experience. It is divided into three parts:

Part I comprises a general survey of the subject, taking up the history, importance, etiology, pathology, clinical history, clinical diagnosis, laboratory diagnosis, prognosis, prophylaxis, and treatment of syphilis.

Part II deals with lues of the different systems of the body, discussing each system separately under the same headings used in Part I of the book, viz.: pathology, clinical history, diagnosis, prognosis, and treatment.

Part III is given over to congenital syphilis, the presentation being similar to that used in Parts I and II for acquired syphilis. The work is not only valuable as a reference book, but is well worth the reading from cover to cover.

